# Supplementary material for: Pilot Production of SARS-CoV-2 Related Proteins in Plants: A Proof of Concept for Rapid Repurposing of Indoor Farms Into Biomanufacturing Facilities
Source: Front Plant Sci. 2020 Dec 23;11:612781. doi: 10.3389/fpls.2020.612781 (PMC7785703; doi:10.3389/fpls.2020.612781)
Supplement: Supplementary file 1 [file Table_1.docx]

Supplementary Material

**Supplementary Table 1.** List of Level 0 plasmids generated and expression vectors used during this work. All sequences can be searched at <https://gbcloning.upv.es/search/features/> using the GB ID.

|  |  |
| --- | --- |
| Name | **GB ID** |
| pUPD2 CR3009 | [GB3576](https://gbcloning.upv.es/feature/GB_UD_A3EB/) |
| pUPD2 CR3018 | [GB3575](https://gbcloning.upv.es/feature/GB_UD_A3ED/) |
| pUPD2 CR3022 | [GB3577](https://gbcloning.upv.es/feature/GB_UD_A3E3/) |
| pUPD2 Sybody3 | [GB3402](https://gbcloning.upv.es/feature/GB_UD_A3E7/) |
| pUPD2 Sybody17 | [GB3403](https://gbcloning.upv.es/feature/GB_UD_A3E9/) |
| pUPD2 Nanobody72 | [GB3404](https://gbcloning.upv.es/feature/GB_UD_A3E5/) |
| pUPD2 natRBD:His | [GB3377](file:///C:/Users/Borja/Downloads/GB3377) |
| pUPD2 bcoRBD:His | [GB3382](https://gbcloning.upv.es/feature/GB3382) |
| pUPD2 His:natRBD:KDEL | [GB3441](https://gbcloning.upv.es/feature/GB3441) |
| pUPD2 His:bcoRBD:KDEL | [GB3442](https://gbcloning.upv.es/feature/GB3442) |
| pUPD2 His:bcoN | [GB3447](https://gbcloning.upv.es/feature/GB3447) |
| pGreen SP-hIgG1 | [GB3604](https://gbcloning.upv.es/feature/VECTOR_IgG1/) |
| pCambiaV1 | [GB1373](https://gbcloning.upv.es/feature/pCambiaV1/) |
| pCambiaV2 | [GB3603](https://gbcloning.upv.es/feature/pCambiaV2/) |
|  |  |
